# Eosinophil-Associated Innate IL-17 Response Promotes *Aspergillus fumigatu*s Lung Pathology

**DOI:** 10.3389/fcimb.2018.00453

**Published:** 2019-01-11

**Authors:** Nathália Luísa Sousa de Oliveira Malacco, Milene Alvarenga Rachid, Isabella Luisa da Silva Gurgel, Tauany Rodrigues Moura, Pedro Henrique Ferreira Sucupira, Lirlândia Pires de Sousa, Daniele da Glória de Souza, Remo de Castro Russo, Mauro Martins Teixeira, Frederico Marianetti Soriani

**Affiliations:** ^1^Centro de Pesquisa e Desenvolvimento de Fármacos, Instituto de Ciências Biológicas, Departamento de Biologia Geral, Universidade Federal de Minas Gerais, Belo Horizonte, Brazil; ^2^Laboratório de Patologia Celular e Molecular, Departamento de Patologia, Instituto de Ciências Biológicas, Universidade Federal de Minas Gerais, Belo Horizonte, Brazil; ^3^Laboratório de Sinalização da Inflamação, Departamento de Análises Clínicas e Toxicológicas, Faculdade de Farmácia, Universidade Federal de Minas Gerais, Belo Horizonte, Brazil; ^4^Laboratório de Interação Microrganismo Hospedeiro, Departamento de Microbiologia, Instituto de Ciências Biológicas, Universidade Federal de Minas Gerais, Belo Horizonte, Brazil; ^5^Laboratório de Imunologia e Mecânica Pulmonar, Departamento de Fisiologia e Biofísica, Instituto de Ciências Biológicas, Universidade Federal de Minas Gerais, Belo Horizonte, Brazil; ^6^Centro de Pesquisa e Desenvolvimento de Fármacos, Departamento de Bioquímica e Imunologia, Instituto de Ciências Biológicas, Universidade Federal de Minas Gerais, Belo Horizonte, Brazil

**Keywords:** Eosinophils role in inflammation, fungal infection, innate immunity, IL-17 innate response, eosinophil lung damage

## Abstract

*Aspergillus fumigatus* is a common widespread microorganism with environmental, biological and clinical relevance. After inhalation, swollen conidia can germinate, colonize and invade pulmonary tissues. Eosinophils have been described as key cells in *A. fumigatus* lung infection. However, their specific role in protecting or damaging lung tissue as well as their relatioship among different *A. fumigatus* strains is poorly understood. Previously, it has been reported that eosinophils are able to produce IL-17 and mediate an innate response that protected mice from infection using Af293 and CEA10 strains. Here, we have developed a set of new experiments with the CEA17-derived A1163 strain of *A. fumigatus*. Using ΔdblGATA1 mice, we demonstrate that eosinophils produce IL-17 and are involved in control of neutrophil, macrophage and lymphocyte recruitment. We found that eosinophils also induce high levels of cytokines and chemokines, generating an intense inflammatory process. Eosinophils are responsible for increased pulmonary dysfunction and elevated lethality rates in mice. Curiously, fungal burden was not affected. To address the role of IL-17 signaling, pharmacological inhibition of this mediator in the airways with anti-IL-17 antibody was able to reduce inflammation in the airways and protect infected mice. In conclusion, our results demonstrate that eosinophils control IL-17-mediated response and contribute to lung pathology after *A. fumigatus* infection. Therefore, eosinophils may represent a potential target for controlling exacerbated inflammation and prevent tissue damage during this fungal infection.

## Introduction

Fungi are common widespread organisms with environmental, biological and clinical relevance. During the last decades, these microorganisms have been found to cause opportunistic diseases with high morbidity and mortality to human beings, especially in patients with defective immunity (Romani, 2008). *Aspergillus fumigatus* is a ubiquitous and saprophytic airborne mold which displays a variety of biological characteristics that confer virulence to mammal hosts (Latgé, 1999; Dagenais and Keller, 2009). After inhalation of airborne conidia, an immunocompetent host is able to manage properly with infection. However, immunocompromised hosts fail to eliminate the fungus, swollen conidia germinate and are able to colonize and invade pulmonary tissues (Dagenais and Keller, 2009; Margalit and Kavanagh, 2015).

*Aspergillus fumigatus* lung infections induce immune responses characterized by T_H_1, T_H_2 and T_H_17 T cells (Cenci et al., 2000; Chaudhary et al., 2010; Werner et al., 2011). There is evidence suggesting that combined T_H_1/T_H_17 deficiency predisposes to fungal diseases (van de Veerdonk et al., 2011; Vinh, 2011). Moreover, Cheng et al. (2010) demonstrated that *Candida albicans* is able to inhibit IL-17 production modulating the Th17 response (Cheng et al., 2010). Additionally, *A. fumigatus* and *C. albicans* can sense IL-17A by GPI (glycosylphosphatidylinositol) anchored proteins and adapt by increasing adhesion and filamentous growth to ensure their persistence (Zelante et al., 2012).

Eosinophils are multifunctional granulocytes implicated in the pathogenesis of numerous inflammatory processes, including airborne fungal infections and allergic diseases. In normal conditions, eosinophils represent a small percentage of circulating leukocytes (1–3% or until a limit of 350 cells/μL of blood in humans), but in response to diverse stimuli, eosinophils are recruited and modulate immune responses through an array of mechanisms (Hogan et al., 2008). In the context of infection, eosinophils are associated with parasite infestations where they possess toxic effects to the pathogen through degranulation and release of different cationic proteins, such as major basic protein (MBP), eosinophil peroxidase (EPO), eosinophil cationic protein (ECP), and eosinophil-derived neurotoxin (EDN) (Hogan et al., 2008; Ravin and Loy, 2015).

There is evidence to suggest that eosinophils play a role in other pathogen infections, including viruses, bacteria and fungi, and also contribute to the development of proper host defense and immunity (Shamri et al., 2011). Eosinophils can recognize pathogens via interaction with complement or pattern recognition receptors (PRRs), including TLRs and dectin-1 (Shamri et al., 2011), these cells can also function as antigen-presenting cells (APCs) (Shi et al., 2000). Eosinophils may function as major effector cells, exacerbating inflammatory processes and inducing tissue damage and dysfunction (Hogan et al., 2008). Conversely, recent studies have suggested that eosinophils might have tissue protective functions (Jacobsen et al., 2007; Lee et al., 2010).

Mucosal fungal diseases may have deleterious effects as a result of an uncontrolled inflammatory process generated by fungus in mammal hosts (Romani, 2008). In such way, immune responses must be timely regulated. This control is crucial for pathogen clearance and healing. The role of eosinophils in initiation and development of non-T_H_2 responses is poorly exploited. Recently, Guerra et al. (2017) described the role of eosinophils in generating an IL-17-mediated protective host response during *A. fumigatus* pulmonary infection (Guerra et al., 2017). Here, using A1163 strain of *A. fumigatus*, one of the strains in which the whole genome was sequenced, we demonstrate that eosinophils actually may play a major contributory role in driving inflammatory host response and disease in a model of acute infection after *A. fumigatus* inhalation. To study the role of eosinophils, we utilized double GATA1 mutant (ΔdblGATA1) mice with complete ablation of eosinophils in the circulation, bone marrow and tissues (Yu et al., 2002).

## Materials and Methods

### Ethics Statement

All animal experiments were approved by the Institution Ethics Committee (Centro de Ética em Experimentação Animal, CETEA/UFMG, Protocol number 62/2011), according to Brazilian national guidelines on animal work (Conselho Nacional de Controle de Experimentação Animal - CONCEA).

### *A. fumigatus* Strain and Culture Conditions

*Aspergillus fumigatus* A1163 strain was used in this study. Culture media used was: complete medium composed of 2% w/v glucose, 0.5% w/v yeast extract and 1x trace elements (YG); and 2% agar (w/v) (Soriani et al., 2008). A1163 strain was grown at 37°C for 48 h. Conidia were harvested by washing the media with 30 mL of sterile phosphate-buffered saline (PBS) and passed through a sterile 40 μm nylon membrane to remove hyphae fragments. Then, conidia were diluted and counted in Neubauer chamber.

### Animal Infections and *in vivo* Abs Administration

In this study, we used male 10–12 weeks old BALB/c (WT) or double GATA1 mutant (ΔdblGATA1) mice, a murine lineage that lodges deletion of the double GATA-binding site (dblGATA) in the GATA-1 promoter and lost the ability to generate eosinophils (Yu et al., 2002). Mice were maintained in pathogen free conditions at Laboratório de Imunofarmacologia (UFMG/Brazil). Prior to infection, mice were anesthetized by steaming up to 3% isoflurane (Biochimico, Brazil) with oxygen and then, were infected intranasally with 1 × 10^8^ conidia of *A. fumigatus* in a total volume of 40 μL of sterile PBS. Infected mice were euthanized 1 and 3 days after infection and bronchoalveolar lavage fluid (BALF) and lungs were harvested. For experiments that antagonize IL-17A, 1 h prior to *A. fumigatus* infection and every day until the euthanasia, WT and ΔdblGATA-1 recipient mice were intranasally injected with 5 μg/mouse of anti–IL-17A neutralizing Ab (kindly provided by Dr. Anne-Gaëlle Besnard and Dr. Bernhard Ryffel, Université d'Orléans and CNRS UMR 7355, Molecular and Experimental Immunology and Neurogenetics, Orléans, France and IIDMM, University of Cape Town, Cape Town, RSA).

### Bronchoalveolar Lavage Fluid (BALF) and Lung Analysis

At indicated time points, infected mice were euthanized with a solution of 180 mg/kg of ketamine and 24 mg/kg of xylazine. Subsequently, BALF was harvested by washing the lungs twice with two aliquots of 1 mL of PBS (Russo et al., 2009). After centrifugation, cell pellets were used for total and differential leukocytes counts. BALF supernatants were used for cytokines, chemokines, and total protein measurements. Protein amounts were quantified in BALF samples using the Bradford assay (Bradford, 1976). After BALF harvesting, lungs were perfused with 5 mL of PBS to remove circulating blood and the right lobes were removed and frozen for subsequent analysis of *myeloperoxidase* (MPO) (Huang et al., 2016), *N-acetyglucosaminidase* (NAG) (Reiner et al., 1981), *eosinophil peroxidase* (EPO) (Strath et al., 1985) as previously described. Also, lungs were harvested to access fungal burden or fixed in formalin 4% to histopathological analysis.

### Flow Cytometry

Leukocytes obtained from BALF were subjected to hypotonic lysis to remove residual erythrocytes, as described previously (Russo et al., 2009). Cells were treated with Fc block (R&D Systems), labeled with relevant mouse antibodies. Namely: Siglec-F - Brilliant Violet 421 (BV421) [BD Biosciences], IL-17 - phycoerythrin (PE) [Biolegend], CD3 – PECy7 [BD Biosciences], CD4 – PE [Biolegend] or FITC [BD Biosciences], RORγT – PerCP [BD Biosciences], TCRγδ – FITC [Biolegend], or isotype control and then fixed with 4% paraformaldehyde. One hundred-thousand events were acquired in a FACScan cytometer. Data were analyzed using FlowJo (Tree Star, Ashland, OR, USA) software. The relevant populations were gated using morphological and surface markers approach. Briefly, we excluded cellular debris and selected high complexity/granularity (SSC) cells. Inside this gate, eosinophils were selected as CD11c^−^ and CD11b^+^ plus Siglec F^+^ cells. After, we analyzed IL-17 production in these cells.

### Cytokine and Chemokine Measurement

Cytokine and chemokine (TGF-β, CXCL1, CCL2, CCL11, TNF-α, IL-1β, IL-12/23p40, IL-17a) levels were quantified in BALF using DuoSet ELISA kits (R&D Systems), in accordance with the manufacturer's instructions.

### Histopathological Analysis

Formalin-fixed left lobes of lungs were dehydrated gradually in ethanol, embedded in paraffin, and 4 μm sections were stained with Hematoxylin and Eosin (H&E) or Grocott's methenamine silver (GMS). The pathology scale considered both the severity and the distribution of morphologic changes within the lungs according to Hubbs et al. (1997). The total pathology score is the sum of the severity and the distribution and potentially range from 0 to 10. The severity scores are: none = 0; minimal = 1; mild = 2; moderate = 3; marked = 4; severe = 5. The distribution scores are: none = 0; focal = 1; locally extensive = 2; multifocal = 3; multifocal and coalescent = 4; diffuse = 5 (Hubbs et al., 1997).

### Assessment of Respiratory Mechanics

Mice were anesthetized with a subcutaneously injection of ketamine and xylazine (8.5 mg/kg xylazine and 130 mg/kg ketamine) to maintain spontaneous breathing under anesthesia. Mice were tracheostomized, placed in a body plethysmograph and connected to a computer-controlled ventilator (Forced Pulmonary Maneuver System®, Buxco Research Systems©, Wilmington, North Carolina USA). This laboratory set-up, specifically designed for mouse, has only a canula volume (death space) of 0.8 mL and provides semi-automatically three different maneuvers: Boyle's Law FRC, quasi-static pressure-volume and fast-flow volume maneuver. First, an average breathing frequency of 160 breaths/min was imposed to the anesthetized animal by pressure-controlled ventilation until a regular breathing pattern and complete expiration at each breathing cycle were obtained, considering Rinx (index of rejection) = 0. Under mechanical respiration the Lung Resistance (Rl) was determined by Resistance and Compliance (RC) test. To measure the Forced Vital Capacity (FVC), the quasi-static pressure-volume maneuver was performed, which inflates the lungs to a standard pressure of +30 cm H_2_O and then slowly exhales until a negative pressure of −30 cm H_2_O is reached. The quasi-static Chord Compliance (Cchord) was calculated with this maneuver considering the volume/pressure of the expiration (from 0 to +10 cm H_2_O). Suboptimal maneuvers were rejected and for each test in every single mouse at least three acceptable maneuvers were conducted to obtain a reliable mean for all numeric parameters (Russo et al., 2018).

### Statistical Analysis

Experiments were performed at least twice and data are presented as the mean ± SD and were analyzed using One-way analysis of variance (ANOVA) followed by Newman-Keuls post-test to compare different groups. Unpaired *t* test was used to compare two groups. Survival analysis was made by Log Rank test. Statistical significance was set as *P* < 0.05 and all graphs and analysis were performed using Graph Pad Prism 5 software.

## Results

### Eosinophil-Deficient Mice Have Decreased Recruitment of Inflammatory Leucocytes to the Airways After Acute Inhalation Infection by *Aspergillus*

**Figure 1:**
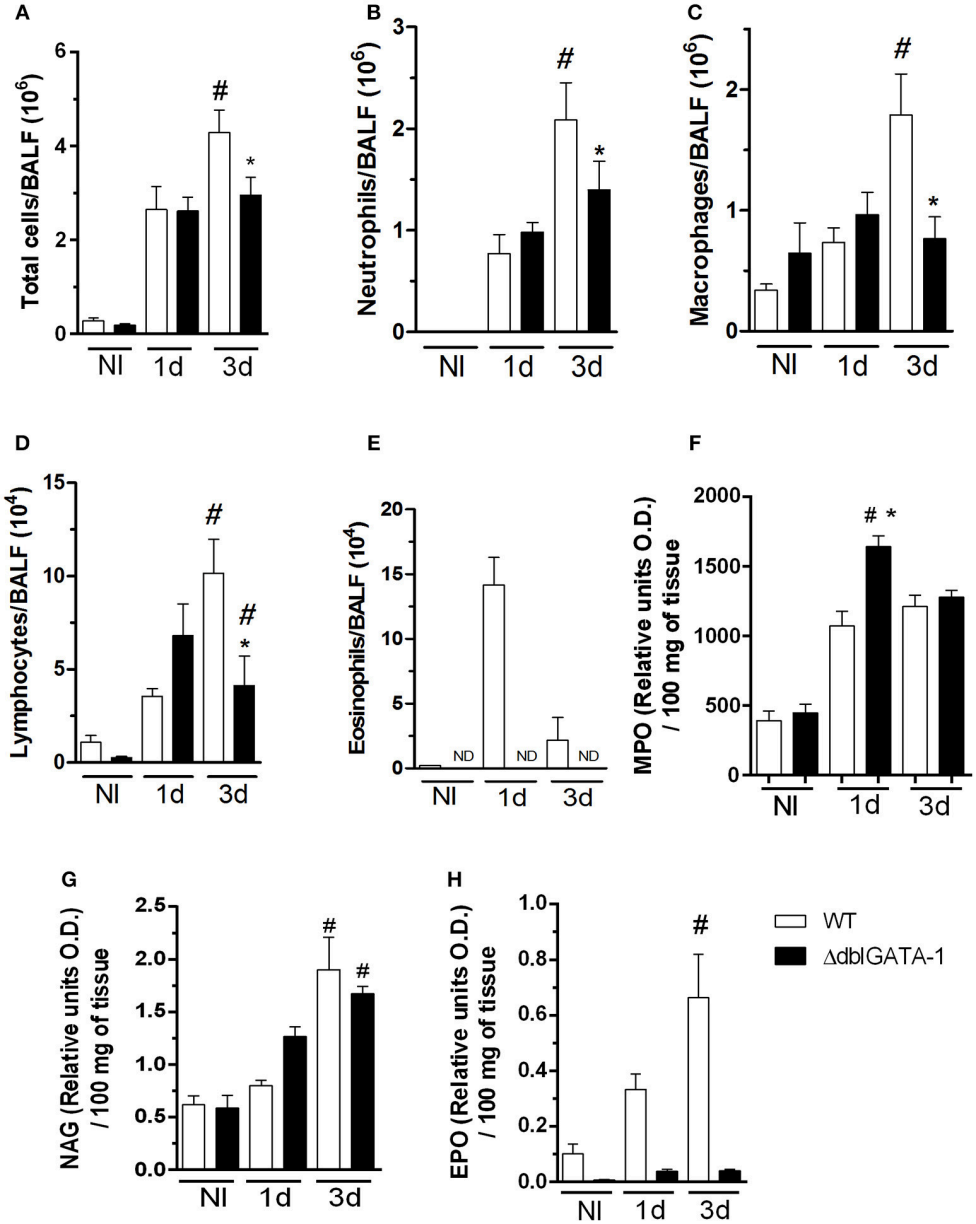
Eosinophil-deficient mice decreased inflammatory cells into airways after *A. fumigatus* infection. WT and ΔdblGATA-1 mice were infected via intranasal with 40 μL of suspension containing 1 × 10^8^ conidia of *A. fumigatus*. BALFs were harvested at 1 and 3 days after infection for inflammatory cell infiltrates determination. Lungs were harvested at 1 and 3 days after infection for NAG, MPO and EPO determination. **(A)** Total cells, **(B)** neutrophils, **(C)** macrophages, **(D)** lymphocytes and **(E)** eosinophils count in BALF. **(F)** MPO **(G)** NAG and **(H)** EPO assays. Data are presented as Mean ± SD (*n* = 5 to 7 mice per group). *Significantly different (*P* < 0.05) compared WT to knockout mice. ^#^Significantly different (*P* < 0.05) between mice with varied times of infection.

In order to investigate the role of eosinophils in acute pulmonary aspergillosis, WT mice were infected intranasally with *A. fumigatus*. There was significant recruitment of leukocytes into the airways of WT infected mice at 1 day of after infection. Leukocyte accumulation reached a peak in the third day after infection. Total leukocyte accumulation in infected ΔdblGATA-1 mice was similar to that of WT mice at day one but significantly lower at day 3 (Figure 1A).

Neutrophils appear to be very important in the context of fungal infections, including *A. fumigatus* (Erwig and Gow, 2016). Our results demonstrate similar number of neutrophils in both WT and ΔdblGATA-1 mice at 1 day after infection. However, at day 3 after infection, there was a decrease of neutrophil migration into the site of infection in ΔdblGATA-1 mice when compared to WT mice (Figure 1B). A similar cell infiltration pattern was observed for macrophages, i.e., there were less macrophages in ΔdblGATA-1 mice at 3 days after *Aspergillus* infection (Figure 1C). Lymphocytes started to accumulate in the airways at day 1 after infection in WT mice with a non-significant increase in ΔdblGATA-1 mice. At day 3 after *A. fumigatus* infection, lymphocyte migration to the airways was decreased in ΔdblGATA-1 compared to WT mice (Figure 1D). The number of eosinophils greatly increased after *Aspergillus* infection in WT mice (Figure 1E). As expected, ΔdblGATA-1 mice had no eosinophils recovered in BALF at any time before or after infection. We also analyzed macrophage, neutrophil and eosinophil infiltration into the lung tissue by measuring tissue NAG, MPO, and EPO contents, respectively. Results demonstrate that tissue levels of NAG and MPO were similar in ΔdblGATA-1 and WT mice after infection (Figures 1F,G), suggesting that the accumulation of these cells in tissues was similar in these animals. Eosinophil levels in lung tissue of WT mice, but not in ΔdblGATA-1 mice, increased at 1 and 3 days after infection (Figure 1H).

### Eosinophils Contribute to Pulmonary Injury and Dysfunction After Acute Inhalation Infection by *Aspergillus*

**Figure 2:**
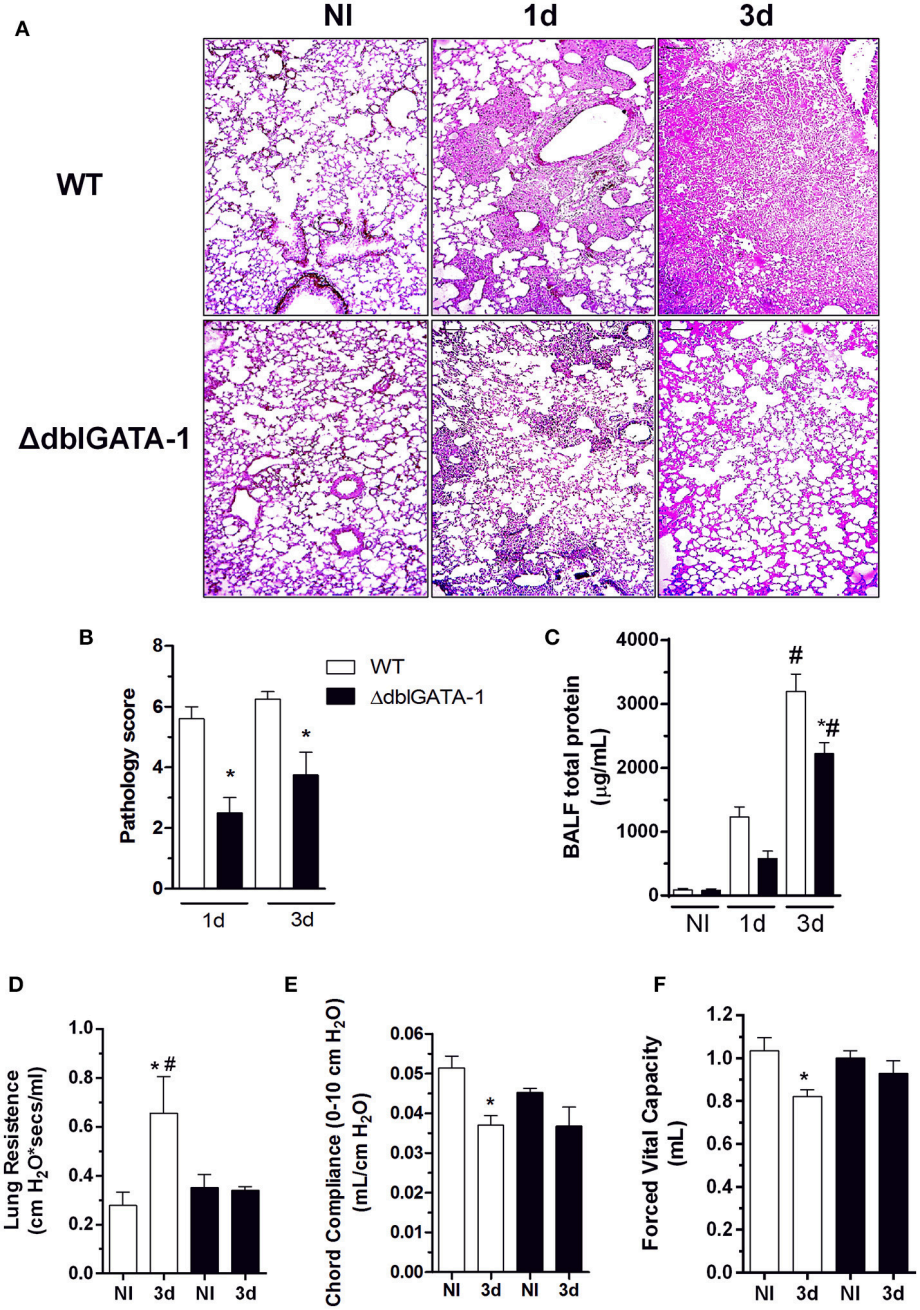
Eosinophil's contribution to lung injury during pulmonary *aspergillosis*. WT and ΔdblGATA-1 mice were infected via intranasal with 40 μL of suspension containing 1 × 10^8^ conidia of *A. fumigatus*. BALF and lungs were harvested at 1 and 3 days after infection. Left lungs were harvested and fixed in formaldehyde 4% and embedded in paraffin. **(A)** Sample sections (*n* = 5 to 7 mice per group) were stained with hematoxilin and eosin and **(B)** pathology score was graded on a 0 to 5-point scale: 0 - no pathological change; 1 - minimal tissue destruction and/or inflammation; 2 - mild tissue destruction and/or inflammation; 3 - moderate tissue destruction and/or inflammation; 4 - marked tissue destruction and/or inflammation; 5 - intense tissue destruction and/or inflammation. **(C)** Total proteins in BALFs was quantified as an indirect measure of vascular permeability. Bars represent 100 μm. Also, forced spirometry was performed to investigate injury by modifications in lung functions after infection. Parameters assessed were lung's ability to streach and expand, by measuring **(D)** RI and **(E)** Cdyn and lung volumes, as presented by **(F)** FVC. Data are presented as Mean ± SD (*n* = 5 to 7 mice per group) *Significantly different (*P* < 0.05) compared WT to knockout mice group. ^#^Significantly different (*P* < 0.05) between mice with different times of infection.

In order to investigate and characterize the course of infection in detail, we analyzed tissue sections of infected mice. Histopathological analysis revealed that the response to infection was characterized by massive leukocyte recruitment into the lungs at day 1 after infection, which became more prominent at 3 days after infection. At day 3, the inflammatory infiltrate covered a large part of the pulmonary parenchyma structure, including alveoli and perivascular regions (Figure 2A). During *A. fumigatus* infection, lungs from WT mice exhibited multifocal to coalescing necrotic and inflammatory areas. Several alveoli and bronchi/bronchioles were filled with neutrophils, macrophages, and lymphocytes (broncopneumonia). Lungs from ΔdblGATA-1 mice displayed significant inflammatory lesions characterized by tissue edema and cellular infiltrates, mainly located around blood vessels and bronchia/bronchiole (Figure 2A). Lesion severity and distribution were quantified and presented as a compound inflammatory score (Figure 2B). Corroborating the results observed in the BALF, inflammatory lesions in infected ΔdblGATA-1 mice were significantly milder than those in WT mice at 1 and 3 days after infection (Figure 2A).

Vascular permeability changes were assessed by measuring the total protein content in BALF. There was a significant increase in vascular permeability changes in the lungs of WT mice infected with the fungus. This was already observed at day 1 post infection and peaked at day 3 after infection. Vascular permeability changes were significantly reduced in infected ΔdblGATA-1 mice (Figure 2C).

In order to evaluate physiological alterations in lung function after acute *A. fumigatus* infection, pulmonary mechanics were evaluated using a forced spirometry technique (Figures 2D–F). In infected WT mice, there was reduction of Forced Vital Capacity (FVC) (Figure 2D). This is consistent with the loss of respiratory area and lung septal thickening observed in the histological analysis. Moreover, we found a decrease in pulmonary elasticity, as measured by Chord Compliance and Lung Resistance (Figures 2E,F, respectively). In agreement with the histopathological score, functional alterations were significantly reduced in the lungs of ΔdblGATA-1 mice after infection.

Taken together, these results indicate that eosinophils contribute to the inflammatory injury and lung dysfunction caused by acute pulmonary *A. fumigatus* infection by A1163 strain in mice.

### Fungal Clearance Is Not Affected by Eosinophils Levels After Acute Infection by *A. fumigatus* Inoculation

**Figure 3:**
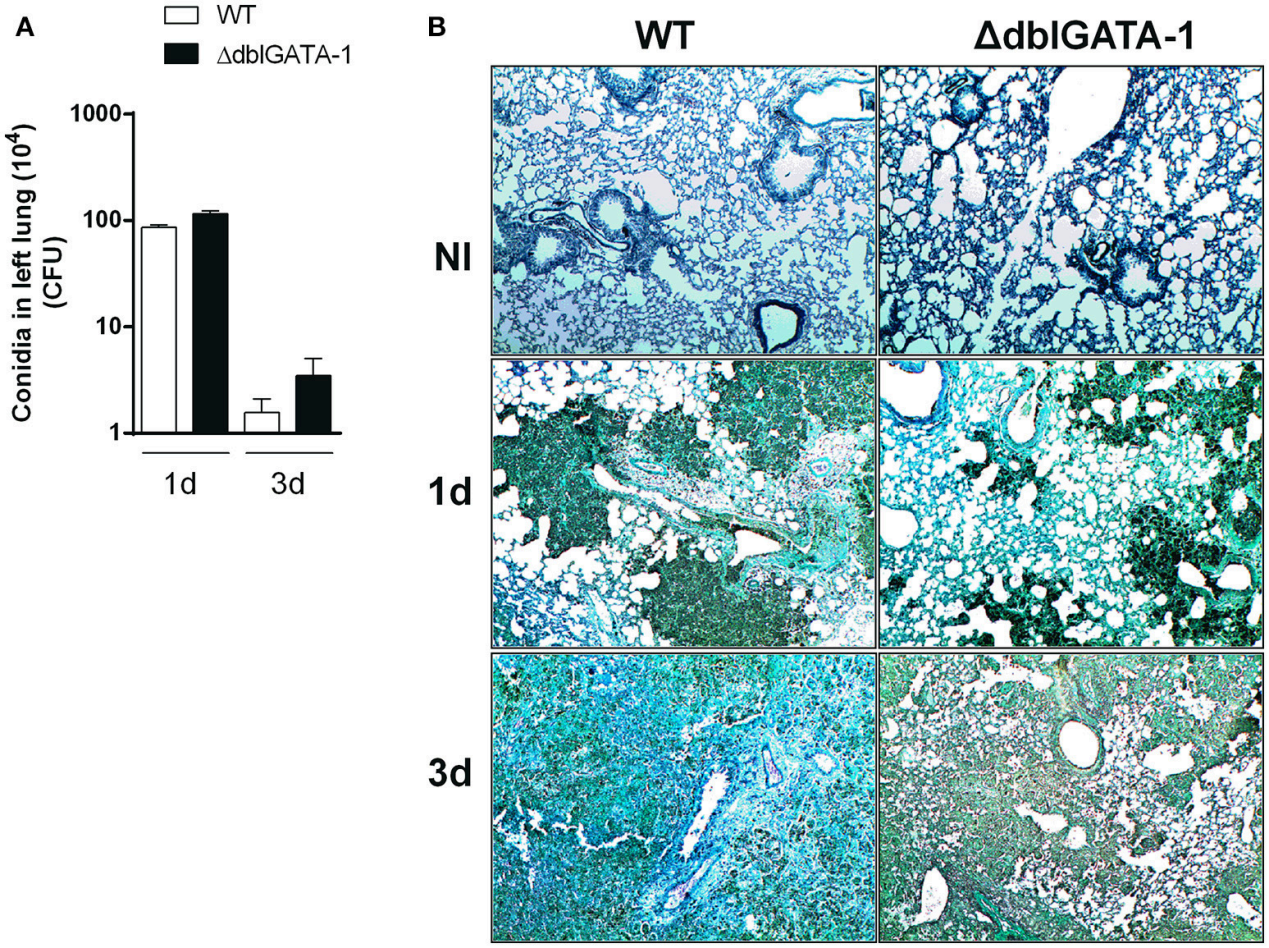
Clearance of *A. fumigatus* into airways from WT and ΔdblGATA-1 mice. WT and ΔdblGATA-1 mice were infected via intranasal with 40 μL of suspension containing 1 × 10^8^ conidia of *A. fumigatus*. Lungs were collected 1 and 3 days after the infection. **(A)** Homogenates from lungs were plated on YG-agar and colony-formit units were counted. **(B)** Lungs were fixed with formaldehyde 4% and embedded in paraffin. Sections were stained with Grocott's methenamine silver. Bars represent 100 μm. Data are presented as Mean ± SD (*n* = 5 to 7 mice per group).

In order to further understand the relevance of eosinophils for the clearance of *A. fumigatus* in the lung, we assessed, by culture of lung homogenates, the number of fungal colony forming units (CFU) at 1 and 3 days after infection. The number of CFU in lungs was greatly elevated at day 1 after infection and significantly decreased at day 3 in infected WT mice. Similar results were obtained in ΔdblGATA-1 mice (Figure 3A). Moreover, we analyzed Grocott's methenamine silver (GMS) staining of lung tissue section, which revealed wide abundance of conidia at 1 day after infection in both WT and ΔdblGATA-1 mice (Figure 3B). Fungal burden was diminished at 3 days after infection, without differences between groups. These results suggest that, although the number of eosinophils increased after *A. fumigatus* infection, these cells seem to play a secondary role in the process of fungal clearance.

### Eosinophils Contribute to Lethality in *A. fumigatus* Acute Pulmonary Infection

**Figure 4:**
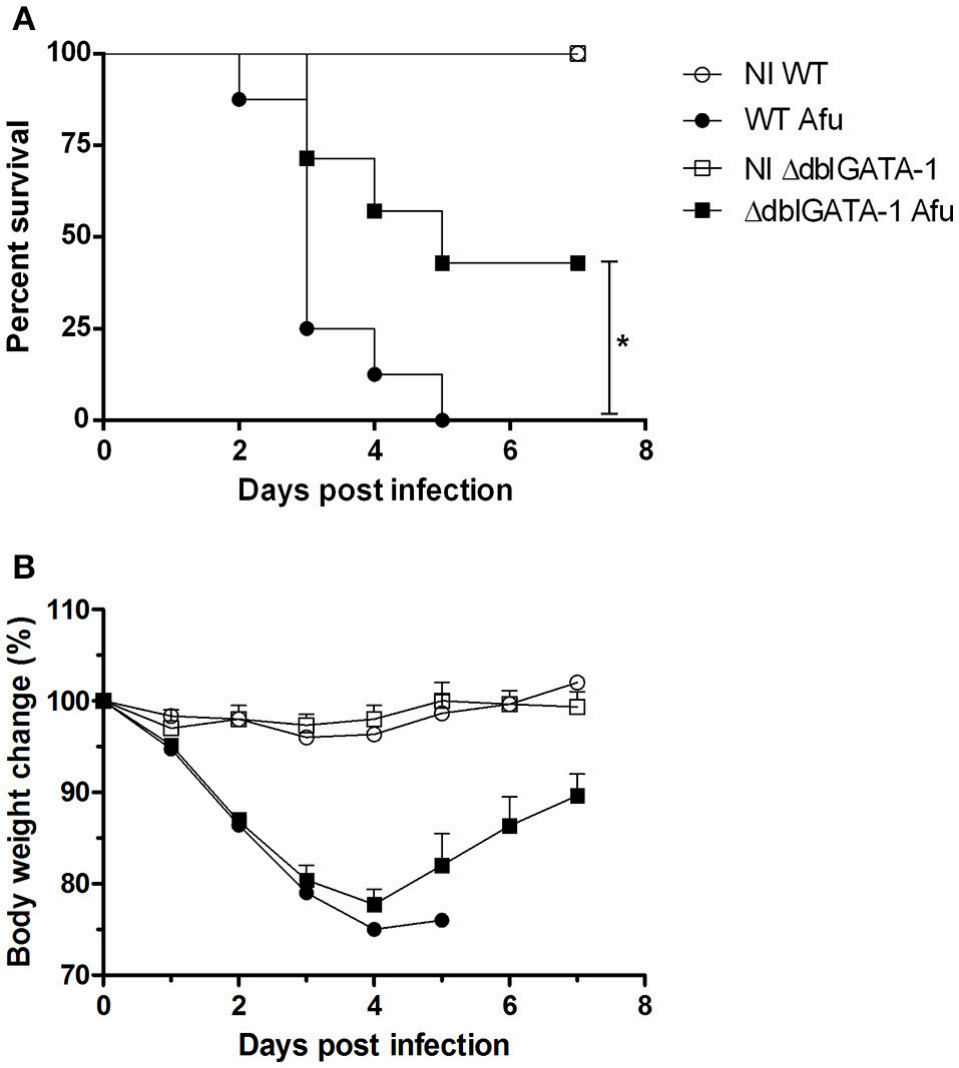
Eosinophils contribute for lethality in *A. fumigatus* acute pulmonary infection. WT and ΔdblGATA-1 mice were infected via intranasal with 40 μL of suspension containing 1 × 10^8^ conidia of *A. fumigatus* and survivor and weight change were monitored for 7 days. **(A)** Comparative lethality and **(B)** body weight change curves of control non-infected group (NI) and infected groups were performed. Data are presented as Mean ± SD (at least 7 mice per group). *Significantly different (*P* < 0.05) compared WT to knockout mice group.

Next, we examined whether there would be any differences in lethality rates and body weight change among WT and ΔdblGATA-1 mice subjected to *A. fumigatus* infection. Animals were intranasally infected with 1 × 10^8^ conidia A1163 strain of *A. fumigatus* and survival and weight loss monitored for 7 days. We observed that WT mice infected with *A. fumigatus* lost significant weight and were highly susceptible to infection. Indeed, all the mice had succumbed to infection at day 5 (Figure 4A). ΔdblGATA-1 mice also lost weight after infection, and this loss was comparable to that observed in WT mice until day 4 after infection. However, a substantial portion of mice recovered their weight loss, resulting in approximately 45% survival at day 7 after infection (Figure 4B).

### Eosinophils Control the Production of Cytokines in Response to *A. fumigatus* Acute Infection

**Figure 5:**
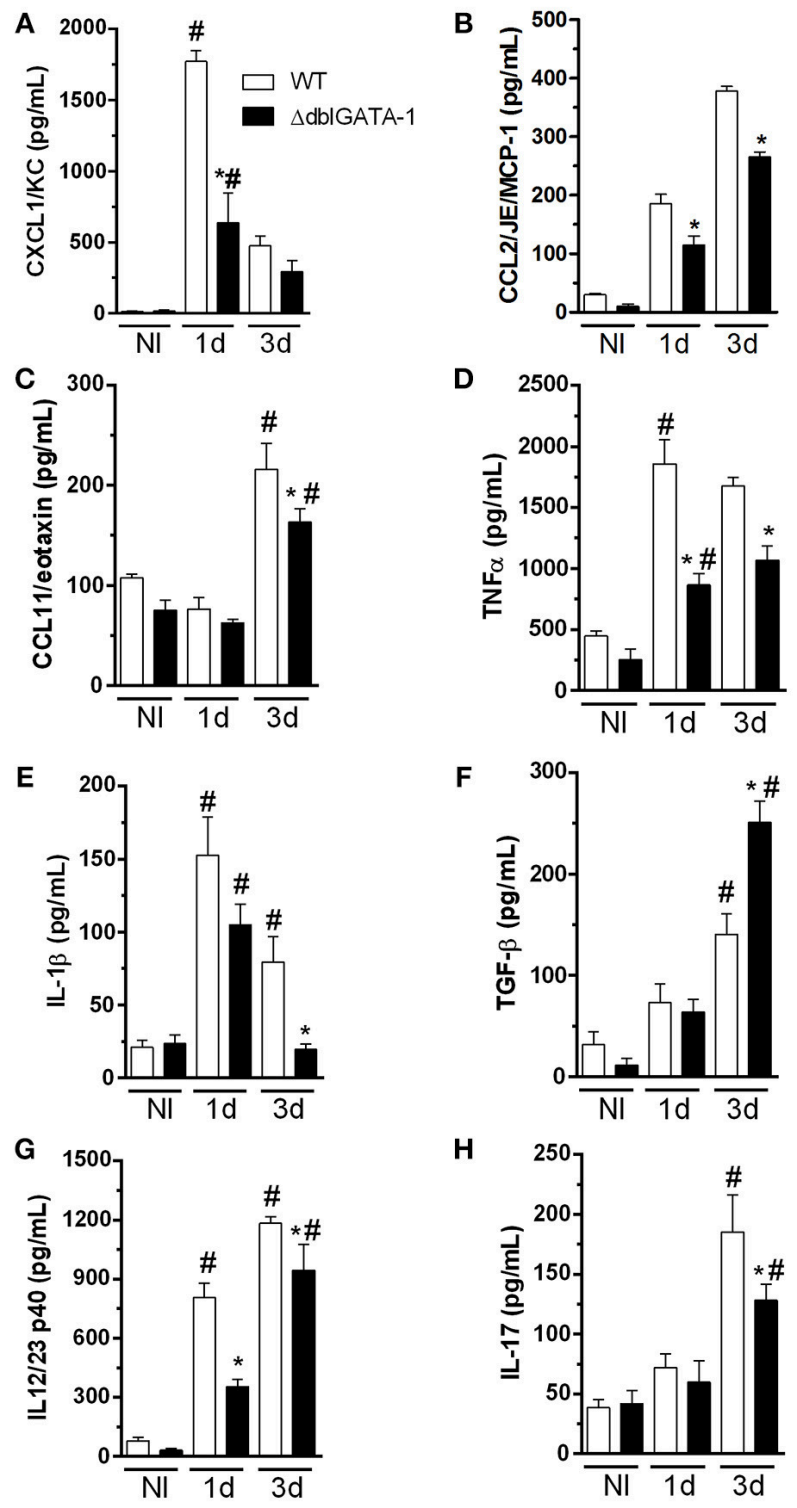
Levels of cytokines and chemokine in BALF during acute *A. fumigatus* infection. WT and ΔdblGATA-1 mice were infected via intranasal with 40 μL of suspension containing 1 × 10^8^ conidia of *A. fumigatus*. BALFs were harvested at 1 and 3 days after infection, centrifuged and stored at −20° C and the mediators were detected by ELISA. **(A)** CXCL1/KC. **(B)** CCL2/JE/MCP-1. **(C)** CCL11/eotaxin. **(D)** TNF-α. **(E)** IL-1β. **(F)** TGF-β. **(G)** IL-12/23 p40. **(H)** IL-17. *Significantly different (*P* < 0.05) compared WT to knockout mice group. ^#^Significantly different (*P* < 0.05) between mice with different times of infection.

Because of the decreased number of recruited cells in BALF and reduced cellular infiltrate in the lungs of ΔdblGATA-1 mice, we evaluated whether levels of inflammatory mediators in BALF supernatants were altered in the absence of eosinophils. We observed that, with the exception of TGF-β, there was a general decrease of chemokines and cytokines levels after *A. fumigatus* infection in ΔdblGATA-1 mice as compared to WT (Figure 5). Indeed, levels of the chemokines CXCL1, CCL2, and CCL11, and the cytokines TNF- α and IL-1β were significantly higher in WT infected mice than uninfected controls. The concentration of these molecules also increased after infection in the lungs of ΔdblGATA-1 mice but the increase was of lower intensity than that observed in WT mice (Figure 5A–E). The concentration of TGF-β increased at day 3 after *A. fumigatus* infection of WT mice. However, in contrast to the other cytokines evaluated, the concentration of TGF-β was greater in the lungs of ΔdblGATA-1 than WT mice (Figure 5F). We also measured IL-12/23 (the p40 subunit) and IL-17A, cytokines relevant for driving Th responses in aspergillosis (Thakur et al., 2015). The concentration of IL-12/23 was elevated at days 1 and 3 after *A. fumigatus* infection, whereas the concentration of IL-17A was only found to be elevated at day 3 after infection (Figures 5G,H). Corroborating results presented by Guerra et al. (2017), IL-12/23 levels were greatly reduced in ΔdblGATA-1 mice at 1 day and at 3 days after *A. fumigatus* inoculation (Figure 5G). Furthermore, ΔdblGATA-1 mice also exhibited a significant reduction in IL-17A levels after 3 days of *A. fumigatus* infection (Figure 5H).

Taken together, these results suggest that eosinophils contribute to overall upregulated secretion of cytokines and chemokines in the airways of *A. fumigatus* infected mice. These cytokines and chemokines are known to contribute to the recruitment of leukocytes to the airways.

### Eosinophils Produce IL-17A and Contribute to a Biased Th17 Response in Acute Infection After *A. fumigatus* Challenge

**Figure 6:**
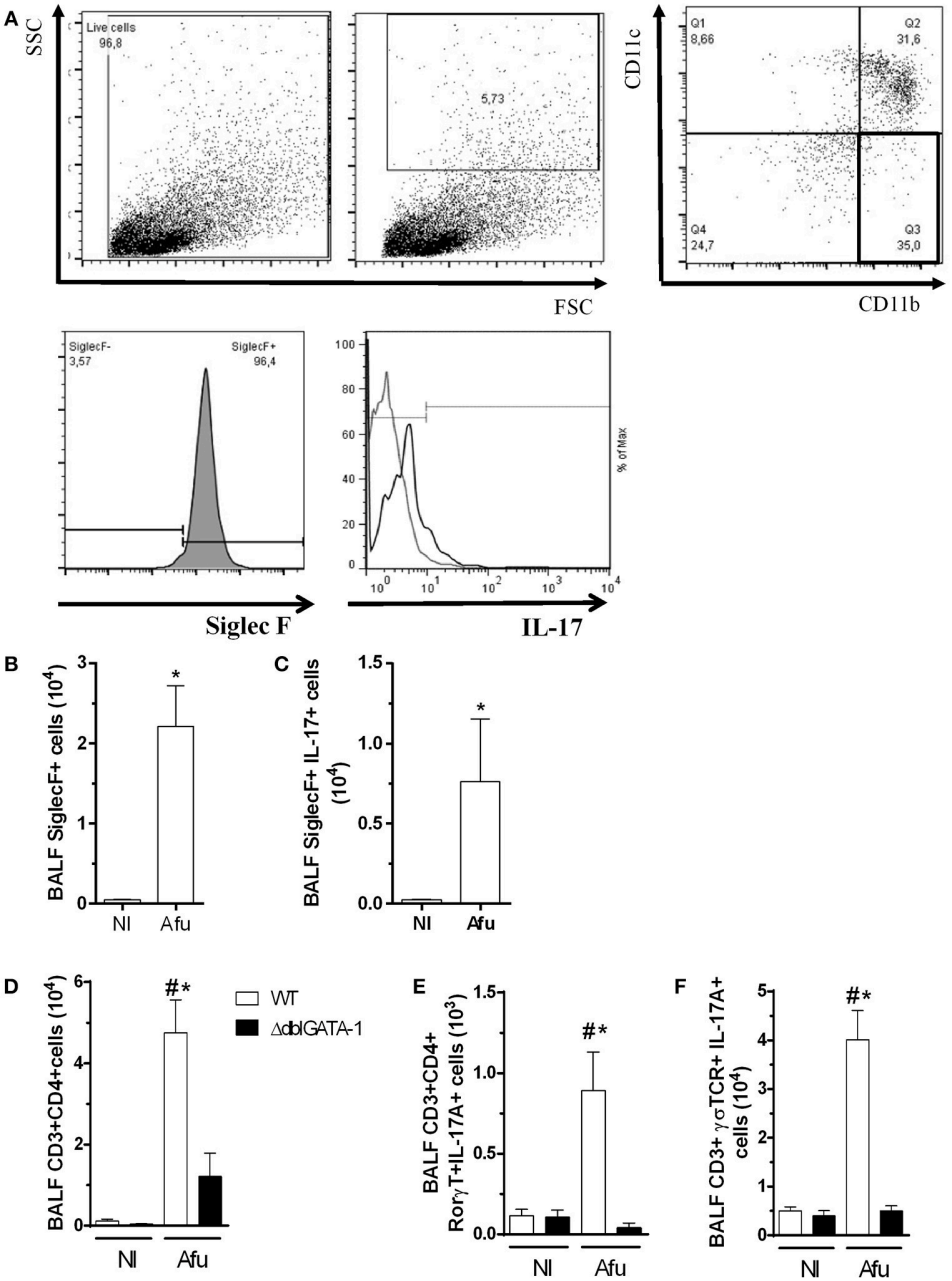
Eosinophils-producing IL-17A contribute for uncontrolled inflammation after acute challenge with *A. fumigatus*. WT and ΔdblGATA-1 mice were infected via intranasal with 40 μL of suspension containing 1 × 10^8^ conidia of *A. fumigatus*. BALFs were harvested at day 3 of infection, centrifuged and the cells were labeled with specific monoclonal antibodies. Samples were measured by flow cytometry and analyzed by FlowJo 7.5.3. The relevant populations were gated using morphological and surface markers approach. **(A)** Gating-strategy and criteria of eosinophils selection. Briefly, we excluded cellular debris and selected high complexity/granularity (SSC) cells. Eosinophils were selected as CD11c^−^ and CD11b^+^ plus Siglec F^+^ cells. After, we analyzed IL-17 production in these cells **(B)** Quantification of Siglec F^+^ cells. **(C)** Siglec F^+^IL-17A^+^ cells. **(D)** CD3^+^ CD4^+^ cells in lymphocytes. **(E)** CD3^+^ CD4^+^ RORγT^+^IL-17A^+^ cells in lymphocytes. **(F)** CD3^+^TCRγδ^+^ IL-17A^+^ cells in lymphocytes. Data are presented as Mean ± SD (*n* = 5 to 7 mice per group). *Significantly different (*P* < 0.05) compared WT to knockout mice group. ^#^Significantly different (*P* < 0.05) between mice with different times of infection.

Because there was decreased IL-17 levels in the airways of infected ΔdblGATA-1 mice, we examined the direct contribution of eosinophils to IL-17A production. We performed a flow cytometry and labeled eosinophils. Analysis of gates revealed a CD11b^+^CD11c-SiglecF^+^ eosinophil population (Figures 6A,B). In addition, we show that among eosinophils, there is a subpopulation of IL-17 positive cells (SiglecF^+^ IL-17^+^; Figure 6C), after *A. fumigatus* lung infection. These results indicate that during *A. fumigatus* acute lung infection, there are eosinophils producing a classical Th-17 marker. Next, we confirmed whether decrease in IL-17A production would be accompanied by decrease in numbers of Th17-producing lymphocytes. In the airways of infected ΔdblGATA-1 mice, there was a reduced number of CD3^+^CD4 ^+^ lymphocytes (Figure 6D) and CD3^+^CD4^+^RORγT^+^IL-17^+^ cells (Figure 6E) when compared to infected WT mice. Moreover, in ΔdblGATA-1 mice, there was also a decrease in the number of innate lymphocytes producing IL-17, defined as CD3^+^TCRγδ^+^IL-17^+^ cells (Figure 6F).

### IL-17A Inhibition in WT Mice Mimicked the Phenotype Observed in *ΔdblGATA-1* and Protected Mice Against *A. fumigatus* Acute Infection

**Figure 7:**
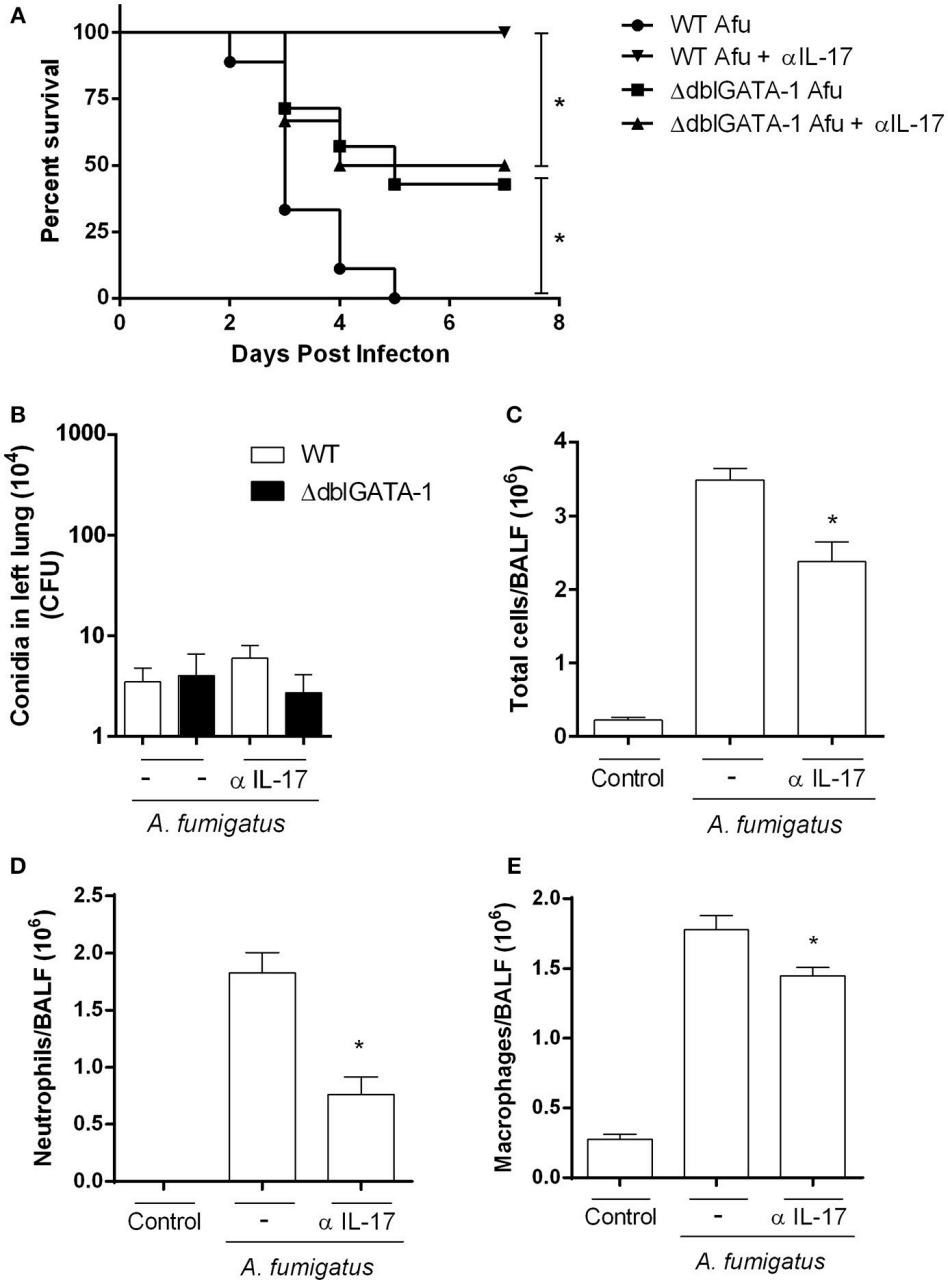
IL-17A inhibition in WT mice recovered the phenotype observed in ΔdblGATA-1 and protected mice against *A. fumigatus* infection. WT mice were intranasally treated with anti-IL-17A Ab (5 μg per mouse) and infected via intranasal with 40 μL of suspension containing 1 × 10^8^ conidia of *A. fumigatus*. **(A)** Lethality curve. After infection, mice were monitored for 7 days and comparative lethality curves were performed (at least 7 mice per group). All WT and ΔdblGATA-1 non-infected mice groups treated or not with anti-IL-17 antibody did not have lethality and were not shown. **(B)** Fungal burden. After 3 days of infection, homogenates from lungs were plated on YG-agar and colony-formit units were counted. **(C–E)** Inflammatory cell infiltrates determination. BALFs were harvested at 3 days after infection and **(C)** Total cells, **(D)** neutrophils and **(E)** macrophages were counted in BALF. Data are presented as Mean ± SD (*n* = 5 to 7 mice per group). *Significantly different (*P* < 0.05) between the same mice group.

Our results have shown that, in the context *A. fumigatus* infection, absence of eosinophils, as observed in ΔdblGATA-1 mice, results in less pulmonary damage and dysfunction. This was associated with decreased IL-17-positive eosinophils and decreased levels of IL-17A. To examine whether this innate IL-17 production was relevant in the model, we inhibited IL-17A in WT mice with an anti-IL-17 antibody and evaluated cellular responses and lethality. Results show that lethality rates were lower in infected WT mice treated with an anti-IL-17 (α-IL-17) antibody (Figure 7A). Treatment of *Aspergillus* infected ΔdblGATA-1 mice with α-IL-17 had no effect on lethality rates. In addition, similar results were observed in CFU counting of WT and ΔdblGATA-1 mice treated with α-IL-17 antibody after infection (Figure 7B). Treatment with anti-IL-17 also reversed the infiltration of leukocytes, neutrophils and macrophages observed in infected WT mice (Figures 7C–E). α-IL-17 treatment had no further effect on the recruitment of leukocytes into the airways of ΔdblGATA-1 infected mice.

## Discussion

In the present study, we demonstrate that a single exposure to *A. fumigatus* results in eosinophil-dependent inflammatory lung injury and dysfunction in immunocompetent mice. Eosinophils are granulocytes well-known for the regulation of inflammation, maintenance of epithelial barrier function, induction of tissue remodeling, and contribution to innate and acquired immunity (Hogan et al., 2008; Shamri et al., 2011). However, because of their cytotoxic actions at sites of infection and inflammation, eosinophils are believed to cause tissue damage in eosinophilic diseases, including asthma and parasitic infections (Fabian et al., 1992; O'Dea et al., 2014).

Using ΔdblGATA1 mice, we show for the first time that eosinophils contribute to IL-17-dependent pulmonary tissue damage and dysfunction during *A. fumigatus* infection. We demonstrate that eosinophils contribute to neutrophil recruitment and this phenotype might be explained by lower production of mediators of inflammation, specifically IL-17. Eosinophil-driven inflammation causes significant lung inflammation, injury, pulmonary dysfunction and consequently, death. Lilly et al. (2014) demonstrated the contribution of eosinophils for the killing of *A. fumigatus* during acute lung infection and also a reduction of inflammatory cytokines into the lungs, especially IL-17 (Lilly et al., 2014). More recently, Guerra et al. (2017) published a set of results suggesting that eosinophils were responsible for controlling the IL-17/IL-23 axis, hence representing important effector and immunomodulatory cells during *A. fumigatus* infection (Guerra et al., 2017). In addition, Fulkerson et al. (2006) have shown that reduction of eosinophilia into the lungs and reduction of Th2 cytokines production were associated with decreased accumulation of other leukocytes in the airways and also smaller cellular infiltrate observed in PAS staining (Fulkerson et al., 2006). In the same way, Fabian et al. (1992) demonstrated that eosinophils stimulated with growth factors seem to have a great contribution in phagocytic activity and antimicrobial response. However, eosinophils also have a role in driving inappropriate inflammatory responses and tissue damage because of their granule constituents such MBP, EPO, ECP, and EDN (Fabian et al., 1992). Here, we demonstrate that the absence of eosinophils interferes with IL-17 production and may affect recruitment of multiple cell types without impacting fungal clearance.

IL-17 effector functions include pivotal roles aiming to maintain mucosal immunity against specific pathogens. These functions are usually related to rapid neutrophil recruitment, stimulation of cell proliferation and induction of inflammatory cytokines and chemokines (Fulkerson et al., 2006). Although IL-17 seems to be protective against extracellular pathogens, this cytokine can also cause injury when it is not tightly regulated (Valeri and Raffatellu, 2016). We corroborated previous results demonstrating the immunomodulatory role of eosinophils in coordinating, together with lymphocytes, a Th17 innate response during *A. fumigatus* infection (Guerra et al., 2017). We also functionally demonstrate that IL-17-producing cells, especially eosinophils, exacerbate the inflammatory response. We inhibited IL-17A with anti-IL-17A antibody and found that this intervention decreased inflammation and lethality rates in WT mice. This result was qualitatively similar to that observed in the absence of eosinophils. Treatment of infected ΔdblGATA-1 mice with anti-IL-17 resulted in no further improvement of the phenotype, suggesting that IL-17 production by eosinophils exacerbates acute inflammation caused by a single exposure to *A. fumigatus*. Although treatment with anti-IL-17 did not alter fungal clearance, this represents a novel therapeutic approach by preventing inflammatory lesions without affect fungal killing.

The diversity in fungal isolates are able to produce different sets of Pathogen-Associated Molecular Patterns (PAMPs) capable of generating immune responses that, at the end, can be responsible for the outcome of infection. Dectin-1, a beta-glucan recognition receptor, has been described as an essential PRR in antifungal immunity during *C. albicans* infection (Taylor et al., 2007). However, Saijo et al. (2007) have demonstrated opposite effects of dectin-1 in immune response against *C. albicans* (Saijo et al., 2007). A few years later, in 2013, Marakalala and cols. showed that the requirement for Dectin-1 in the control of systemic *C. albicans* infections is fungal strain-specific (Marakalala et al., 2013).

Moreover, O'Dea et al. (2014) showed that repeated instillations of the high chitin-expressing isolate, Af5517, augmented airway eosinophilia and Th2-related chemokines and cytokines in the lungs of mice when compared to those induced by the Af293 strain (O'Dea et al., 2014). Another study demonstrated that the increase of chitin in *A. fumigatus* surface, induced by caspofungin, was related to eosinophil recruitment into the lungs in neutropenic mice with aspergillosis and elevated fungal burden and disease severity compared to eosinophil deficient mice (Amarsaikhan et al., 2017). Considering that differences in virulence among fungal lineages and species are poorly understood, the knowledge of the molecular mechanisms by which diverse immune and inflammatory responses occur against *A. fumigatus* will improve our understanding related to the outcome of infection by different strains.

In fungal infections, variations due to strain isolates, inoculum, and site of infection may represent key points related to fungal growth, colonization, invasion and sensitivity to antifungal agents, hence affecting the outcome of infection. It has been described that there are key physiological differences among strains that are able to cause diverse immune and inflammatory responses, as well as disease severity variability. For instance, two common isolate strains, Af293 and CEA10, displayed significant differences in physiological responses to environmental stimuli, pathogenic potential and virulence in a C57 murine model of Invasive Pulmonary Aspergillosis (IPA). While Af293 lineage was considered an inflammatory strain, with induction of IFN-γ, IL-10, and IL-1β release, the CEA10 lineage was considered hyper inflammatory, inducing a great amount of IL-17 release (Rizzetto et al., 2013). Caffrey-Carr et al. (2017) also demonstrated that the heterogeneity of *A. fumigatus* isolates can result in a distinct inflammatory response. However, the exactly way the differences among isolates affect immune response, lung damage and outcome of the disease is not clear (Caffrey-Carr et al., 2017). These works point to the importance of studying *A. fumigatus* isolates to complete understand factors that may contribute to different outcomes of the disease and the pursuit for novel therapeutic targets.

In the current study, we evaluated immune responses induced by *A. fumigatus* A1163 strain, which is a uracil auxotrophic derivative of *A. fumigatus* CEA17 isolate (CEA10 derivative) and converted to pyrG^+^ via *A. niger* pyrG gene ectopic insertion (Fedorova et al., 2008). The genome of A1163 strain has been sequenced and compared with the Af293 parental strain and this analysis revealed core, variable and about 2% unique genes in each genome. A great number of Af293- and A1163-specific genes contain pseudogenes and repeated elements that also appear to be involved in important cellular functions, such as secondary metabolism, detoxification, heavy metal and osmotic stress. These cellular functions can facilitate adaptation to different environments such as soil and mammalian hosts (Fedorova et al., 2008). More studies need to be performed for a better understanding of host-pathogen interactions and their relation with strain-dependent differences in immune and inflammatory response, since this is a limitation in our study.

Taken together, our data demonstrate that eosinophils promote detrimental pulmonary inflammation in response to *A. fumigatus* infection by acting as a source of IL-17. Moreover, it is clear that different genetic backgrounds of the pathogen, like chitin metabolism and composition of the cell wall, may be able to generate diverse outcomes. More studies are required to further understand the role of eosinophils during *Aspergillus* infection and how the physiological fungal diversity, such as carbohydrates of the cell wall, among others, are important for the establishment of an adequate immune response.

## Author Contributions

FS conception of the study. NM, RR, and FS designed the experiments. NM, IG, TM, RR, PS, and MR performed the experiments. NM, IG, LS, RR, DS, MT, and FS interpretation of the results and data analysis. MR, DS, MT, and FS contributed reagents, materials, and analysis tools. NM, MT, and FS wrote the manuscript. All authors read and approved the final manuscript.

### Conflict of Interest Statement

The authors declare that the research was conducted in the absence of any commercial or financial relationships that could be construed as a potential conflict of interest.
